# Supplementary material for: A first survey for herbicide resistant weeds across major maize growing areas in the North Island of New Zealand
Source: PLoS One. 2024 Mar 7;19(3):e0299539. doi: 10.1371/journal.pone.0299539 (PMC10919694; doi:10.1371/journal.pone.0299539)
Supplement: Attachment — Submitted filename: Ngow et al Reviewers comments.docx [file pone.0299539.s002.docx]

**A first survey for herbicide resistant weeds across major maize growing areas in the**

**North Island of New Zealand**

*Overall comments*

Was there the need for keeping the seed collected from the individual plants separate as in the text the results and discussion refer only to the percentage of farms with resistance

*Introduction*

Line 43	I always query the term of ‘over-use’ of herbicides in relation to the evolution of resistance.	When many herbicides were first released herbicide resistance was not discussed so how can something be over-used if it is the best method of achieving something. In hindsight yes but with the available knowledge at the time no.

Line 57	Were any non-random surveys done elsewhere as well? Line 60-61 basically repeats Line 57-58 but adds the non-random element.

Line 64	This sentence doesn’t really fit anywhere. Maybe add this to end of sentence Line 68 ‘…maize in New Zealand as nor systemic random surveys have been conducted’

*Materials and Methods*

Line 83-4	This sentence is not required, it states the obvious.

Line 91	Change ‘up-to’ to ‘up to’

Line 95-104	What time of year? Did the daylength equate to that of the weeds normal growing season?

Line 108	Was this a flat fan nozzle (designed for overlapping nozzles) or an even flat nozzle (banding) as a single flat fan nozzle will have different application rates across the width of the spray band without the overlap from extra nozzles.

Line 110	Move Table so that it is not across two pages

Line 116	Why was three chosen? Did this vary if below 20 seeds germinated. Resistance is commonly defined as greater than 20% survival, why wasn’t this definition used for consistency with other research?

*Results*

Line 124-6	*C. album,* *D. sanguinalis* and *E. crus-galli* don’t need their genus spelt out as they have already had their full name written in the text

Line 136	Same as above for *D. sanguinalis* and *E. crus-galli*

Table 2	The difference between farms and samples need to be better explained. As Line 86-7 states that one paddock per farm was sampled, therefore Line 91 needs to define whether the seeds from each individual plant were kept separate (as per other NZ surveys) or bulked (as per surveys in other countries). This would explain how samples tested are greater than farms sampled.

Line 142	Delete ‘mean = 31%’ in brackets as already stated in the text

Line 144	Mean of Bay of Plenty farms not provided, just says quarter while for Waikato it says both half and (50%)

Line 144-5	What is the CI for *C. album*?

Line 150	*F. convolvulus* doesn’t need the genus spelt out as full name has already been written

Table 3&4	Remove second column ‘Farms surveyed’ as this just repeats for all species . Mention this at Lines 134-5 in same brackets as number of samples eg (Waikato 36 farms: 305 samples)

Line 162	*E. crus-galli*

*Discussion*

Line 182	Better define this survey rather than ‘arable’ was it ‘wheat and barley farms’

Line 183	Change ‘weeds’ to ‘weed species’ and ‘and although’ to ‘except for C. *album* and *D. sanguinalis*.’ Start new paragraph with ‘Atrazine …’. Currently the flow of this paragraph goes from 40% resistance, not resistant, widespread resistance, this is confusing and loses the message of although resistance is common it is only in two species.

Line 194	add ‘reported previously’ and references to this sentence

Line 202-5	Move these two sentences to the first paragraph of the discussion and merge with the sentence ‘Most weed species ...’ (as suggested) as this is an important difference between these two surveys and needs to be given higher priority in the discussion

*References*

1 delete ‘:a dissertation submitted in partial fulfilment of the requirements for the degree of Masters [i.e. Master] of Applied Science at Lincoln University’ and replace with ‘MAppSc Thesis’
2 remove capitals from ‘survey of maize areas and volumes’
3 Add capitals ‘Pest Management Science’

6	as per reference 2

7	as per reference 2

15	as per reference 2

19	as per reference 2, full Journal name

24	change to ‘Pesticide Resistance’

25	full Journal name

26	Is the editor’s name required in this reference?
