# Supplementary material for: A first survey for herbicide resistant weeds across major maize growing areas in the North Island of New Zealand
Source: PLoS One. 2024 Mar 7;19(3):e0299539. doi: 10.1371/journal.pone.0299539 (PMC10919694; doi:10.1371/journal.pone.0299539)
Supplement: Attachment — Submitted filename: Response to Reviewers.docx [file pone.0299539.s003.docx]

**Authors’ response to reviewers’ comments on: A first survey for herbicide resistant weeds across major maize growing areas in the North Island of New Zealand**

*Overall comments*

Was there the need for keeping the seed collected from the individual plants separate as in the text the results and discussion refer only to the percentage of farms with resistance.

**Response:** We made addition of this text to the second paragraph under the subheading ‘Sample collection’: “Our goal to detect the presence of resistance, not to characterize the proportion of resistance in individual fields. Collecting from individual plants means that the seeds collected share a genetic history (from the ‘mother’) and a larger proportion of the seed sample is likely to contain resistant traits. This approach also allowed us to detect any possible cross-resistance or multiple resistance where plants share a common genetic background.”

*Introduction*

Line 43	I always query the term of ‘over-use’ of herbicides in relation to the evolution of resistance.	When many herbicides were first released herbicide resistance was not discussed so how can something be over-used if it is the best method of achieving something. In hindsight yes but with the available knowledge at the time no.

**Change:** Growers of maize rely heavily on herbicides to control weeds. Across the world, repeated use of herbicides within a single mode of action has led to the repeated evolution of herbicide resistant weeds [3].

Line 57	Were any non-random surveys done elsewhere as well? Line 60-61 basically repeats Line 57-58 but adds the non-random element.

**Change:** Reworded to remove repetition. To answer the question: investigations in the past of resistance did include sites around farms with resistance, but these were not done in a randomized, systematic way (see James et al. 2005, Rahman & Patterson 1987 and Rahman et al. 1983). We also tested some samples collected ‘outside’ of the survey (which were not included in the manuscript).

Line 64	This sentence doesn’t really fit anywhere. Maybe add this to end of sentence Line 68 ‘…maize in New Zealand as nor systemic random surveys have been conducted’

**Change:** Suggested changes made, sentence deleted.

*Materials and Methods*

Line 83-4	This sentence is not required, it states the obvious.

**Change:** Removed as suggested.

Line 91	Change ‘up-to’ to ‘up to’

**Change:** Suggested changes made.

Line 95-104	What time of year? Did the daylength equate to that of the weeds normal growing season?

**Change:** Spring-autumn. Added text: “Plants were grown during their normal growing season between spring and autumn, with the majority planted in November.”

**Additional change**: Subheading added for plant propagation, moved herbicide treatment to the precede the appropriate section.

**Additional change** **- added the bolded text:** Seed samples were stored at 5°C for **at least a month** before being planted.

Line 108	Was this a flat fan nozzle (designed for overlapping nozzles) or an even flat nozzle (banding) as a single flat fan nozzle will have different application rates across the width of the spray band without the overlap from extra nozzles.

**Response**: Sentence added and clarified that it is a “flat” fan nozzle. “The moving belt and trays or pots are positioned in the central third of the fan area to ensure even coverage.”

Line 110	Move Table so that it is not across two pages.

**Response**: The table (and other tables) now do not cross more than one page.

Line 116	Why was three chosen? Did this vary if below 20 seeds germinated. Resistance is commonly defined as greater than 20% survival, why wasn’t this definition used for consistency with other research?

**Response:** We clarified germination criteria for inclusion in the text. We used the same threshold in our earlier survey work, and we think this threshold is appropriate as we were not bulking samples, which is also different from the bulking approach used in Australia.

**Change:** *In the second paragraph under the heading “Herbicide treatment” we added*: “Similar to other studies [16,17], including our earlier survey [5] we use a threshold to classify samples, those with fewer than three seedlings surviving treatment were not classified as resistant. This choice is appropriate for un-bulked samples. Seed samples without sufficient germination (less than 4) were classified as ‘collected’ but not ‘tested’.”

*Results*

Line 124-6	*C. album,* *D. sanguinalis* and *E. crus-galli* don’t need their genus spelt out as they have already had their full name written in the text

**Change:** Suggested changes made

Line 136	Same as above for *D. sanguinalis* and *E. crus-galli*

**Change:** Suggested changes made

Table 2	The difference between farms and samples need to be better explained. As Line 86-7 states that one paddock per farm was sampled, therefore Line 91 needs to define whether the seeds from each individual plant were kept separate (as per other NZ surveys) or bulked (as per surveys in other countries). This would explain how samples tested are greater than farms sampled.

**Response**: clarification added to methods section under subtitle “Sample collection” and also to table caption.

Line 142	Delete ‘mean = 31%’ in brackets as already stated in the text

**Change:** Suggested changes made

Line 144	Mean of Bay of Plenty farms not provided, just says quarter while for Waikato it says both half and (50%)

**Response:** It was exactly half and a quarter for those but added means to clarify.

Line 144-5	What is the CI for *C. album*?

**Change:** Added CI for proportion of resistant over tested *C. album* seed samples and proportion of farms with *C. album* resistant over tested. Also added this for *D. sanguinalis* in its section.

Line 150	*F. convolvulus* doesn’t need the genus spelt out as full name has already been written

**Change:** Suggested changes made

Table 3&4	Remove second column ‘Farms surveyed’ as this just repeats for all species . Mention this at Lines 134-5 in same brackets as number of samples eg (Waikato 36 farms: 305 samples)

**Change:** Column removed. The number of farms sampled was specified in the materials and methods and also in Table 2 (farms surveyed). I didn’t add that as it may make that sentence too complicated.

Line 162	*E. crus-galli*

**Change:** Suggested changes made

*Discussion*

Line 182	Better define this survey rather than ‘arable’ was it ‘wheat and barley farms’

**Response:** Mostly but not all. Arable used here. It included small numbers of other species grown for seed multiplication, e.g., clover, ryegrass (also wheat and barley is often the current crop preceded by other crop rotations.

Line 183	Change ‘weeds’ to ‘weed species’ and ‘and although’ to ‘except for C. *album* and *D. sanguinalis*.’ Start new paragraph with ‘Atrazine …’. Currently the flow of this paragraph goes from 40% resistance, not resistant, widespread resistance, this is confusing and loses the message of although resistance is common it is only in two species.

**Change:** Suggested changes made.

Line 194	add ‘reported previously’ and references to this sentence

**Change:** Suggested changes made.

Line 202-5	Move these two sentences to the first paragraph of the discussion and merge with the sentence ‘Most weed species ...’ (as suggested) as this is an important difference between these two surveys and needs to be given higher priority in the discussion

**Response:** Suggested changes made.

*References*

1 delete ‘:a dissertation submitted in partial fulfilment of the requirements for the degree of Masters [i.e. Master] of Applied Science at Lincoln University’ and replace with ‘MAppSc Thesis’
2 remove capitals from ‘survey of maize areas and volumes’
3 Add capitals ‘Pest Management Science’

6	as per reference 2

7	as per reference 2

15	as per reference 2

19	as per reference 2, full Journal name

24	change to ‘Pesticide Resistance’

25	full Journal name

26	Is the editor’s name required in this reference?

**Response:** suggested changes made: replaced the text with MAppSc Thesis, fixed capitalization errors, removed the editor’s name. Additionally: italicized binomials.

A comment: In your Methods section, please provide additional information regarding the permits you obtained for the work. Please ensure you have included the full name of the authority that approved the field site access and, if no permits were required, a brief statement explaining why.

**We added the text in bold:** Stakeholders were contacted in randomised order, **to obtain permission for field sampling**, from two databases to provide a total 70 randomly selected farms, 36 from Waikato, 16 from Bay of Plenty, 11 from Hawke’s Bay and 7 from Wellington. **Farmers were contacted by phone again to confirm their permission on the day we visited their farms.**

A comment: We note that Figure 1 in your submission contain [map/satellite] images which may be copyrighted. All PLOS content is published under the Creative Commons Attribution License (CC BY 4.0), which means that the manuscript, images, and Supporting Information files will be freely available online, and any third party is permitted to access, download, copy, distribute, and use these materials in any way, even commercially, with proper attribution. For these reasons, we cannot publish previously copyrighted maps or satellite images created using proprietary data, such as Google software (Google Maps, Street View, and Earth). For more information, see our copyright guidelines: <http://journals.plos.org/plosone/s/licenses-and-copyright>.

**Response:** We added the attribution: “Map tiles by Stamen Design, under CC BY 4.0. Contains information from OpenStreetMap and OpenStreetMap Foundation, which is made available under the Open Database License.”

A comment: Please include captions for your Supporting Information files at the end of your manuscript, and update any in-text citations to match accordingly. Please see our Supporting Information guidelines for more information.

**Response:** I have included a caption as according to the guidelines: “**S1 Table: Herbicide resistance screening data.** This data is from weed seed samples collected from North Island, New Zealand maize crops.”
