# Supplementary material for: A first survey for herbicide resistant weeds across major maize growing areas in the North Island of New Zealand
Source: PLoS One. 2024 Mar 7;19(3):e0299539. doi: 10.1371/journal.pone.0299539 (PMC10919694; doi:10.1371/journal.pone.0299539)
Supplement: Attachment — Submitted filename: ReviewPLOSONE.2024.01.19.docx [file pone.0299539.s004.docx]

Review of A first survey for herbicide resistant weeds across major maize growing areas in the North Island of New Zealand

January 2024

Overview: The authors have addressed all of the points in the first review and made good improvement in the content and clarity of the manuscript. There are just a few notes I would add to make this publication ready. Thanks to the authors for their contribution of a very interesting manuscript and careful attention to reviewer suggestions.

Line specific notes

In the response to reviewers in the materials and methods section, the authors indicated that a new subheading was added for ‘ plant propagation’. This cannot be found. Everything makes sense without it, but if there is a heading or a section missing by accidental deletion, this should be included.

Line 92 The term ‘bulking’ or ‘bulked samples’ is introduced for the first time. The meaning is clear, but I wonder if this is really the most accurate or widely used term in the literature for such methods. It seems like a regional slang.

Table 2. While the description has been improved, the detail from line 93 should be included to help understand when the table stands on its own. Otherwise the description is still unintelligible.

Table 4. Entire table in a different font. The description for the heading of the current column 2 is confusing between Table 3 and 4. Both should have the same language and the description should clarify what is meant by ‘farms’, ‘samples’, ‘paddocks’ or ‘fields’, similar to including the line 93 in Table 2, so that the table can stand on its own.
