# Supplementary material for: A first survey for herbicide resistant weeds across major maize growing areas in the North Island of New Zealand
Source: PLoS One. 2024 Mar 7;19(3):e0299539. doi: 10.1371/journal.pone.0299539 (PMC10919694; doi:10.1371/journal.pone.0299539)
Supplement: Attachment — Submitted filename: Response to Reviewers.docx [file pone.0299539.s005.docx]

**Authors’ response to reviewers’ comments on: *A first survey for herbicide resistant weeds across major maize growing areas in the North Island of New Zealand***

Here are our changes in response to the reviewers’ comments.

**A comment:**

In the response to reviewers in the materials and methods section, the authors indicated that a new subheading was added for ‘ plant propagation’. This cannot be found. Everything makes sense without it, but if there is a heading or a section missing by accidental deletion, this should be included.

*Change:* We have put the subheading back. *It may have accidentally been deleted, possibly due to merging tracked changes.*

**Line 58**

Sentence feels ‘clumsy’. Maybe ‘no random surveys to determine the proportion of farms with resistant weeds were conducted anywhere in New Zealand.’ Is this all crops or just maize?

*Change:* Removed that sentence and the next. Added a sentence on line 55: *“Despite there being several documented cases of resistance, no systematic survey for resistant weeds in New Zealand maize crops has been conducted.”* The paragraph formerly beginning on line 57 is now merged with the previous. Removed two sentences from that paragraph reiterating the previous assumption of resistant weed distribution. Order of sentence changed to: “*The current lack of knowledge regarding the prevalence of these resistant biotypes, including the recently discovered nicosulfuron-resistant D. sanguinalis, motivated an investigation of the prevalence of resistant weeds in maize.”*

*To answer the question*: No random surveys for resistance had in fact been done before this in New Zealand. Between 2019-2023, the first surveys for resistance began in cereals, maize and vineyards. However, when a new case of resistance was detected, there were smaller local investigations of nearby farms (for instance studies of dicamba-resistant *C. album* included multiple farms from eastern Waikato). These studies did not describe the prevalence of those resistant weeds.

**Line 72**

*An additional change:* We changed a sentence, for clarity, to: *“We anticipated that similar detections of new resistant weed species could be observed in surveys for resistance* *in maize.”*

**Line 92**

The term ‘bulking’ or ‘bulked samples’ is introduced for the first time. The meaning is clear, but I wonder if this is really the most accurate or widely used term in the literature for such methods. It seems like a regional slang.

*Change:* Removed the word ‘bulked’; now the sentence reads: “*Seed samples were collected from individual weeds (not combined across multiple plants) within surveyed paddocks; seed from up to ten plants per species were collected from per field, at distances of at least 20 metres apart.*” Additionally, changed a sentence on line 127 to: “*This choice is appropriate for samples collected from individual plants.”*

**Table 2.**

While the description has been improved, the detail from line 93 should be included to help understand when the table stands on its own. Otherwise the description is still unintelligible.

*Change:* Added to the table caption: “***Count of farms and seed samples of weed species that were tested.*** *This omits sites with samples that did not germinate. Seed samples were collected from individual plants within farms.*”

**Table 4.**

Entire table in a different font. The description for the heading of the current column 2 is confusing between Table 3 and 4. Both should have the same language and the description should clarify what is meant by ‘farms’, ‘samples’, ‘paddocks’ or ‘fields’, similar to including the line 93 in Table 2, so that the table can stand on its own.

*Change:* Standardized font to Times New Roman. Labelled the first column in both Table 3 and 4 as ‘*Farms Tested*’. Added sentence to captions on Table 3 and 4: “*The number of farms tested are those where seed samples of that weed species were collected and tested with herbicides.*” All mentions of ‘paddocks’ have been changed to ‘fields’ for standardization. Two mentions of ‘fields’ were corrected to ‘farms’ in the descriptions of Table 3 and 4.

**Reference 3**

use capitals for journal name Pest Management Science

*Change:* Corrected to capitals.

**Reference 17**

remove abbreviation Crop and Pasture Science not Crop Pasture Sci

*Change:* Corrected to Crop and Pasture Science.

**An additional change:**

We have changed the order of the authors to better reflect their contributions: Zachary Ngow, Trevor K. James, Ben Harvey and Christopher E. Buddenhagen
